# Strong Association of a Common Dihydropyrimidine Dehydrogenase Gene Polymorphism with Fluoropyrimidine-Related Toxicity in Cancer Patients

**DOI:** 10.1371/journal.pone.0004003

**Published:** 2008-12-23

**Authors:** Eva Gross, Birgit Busse, Matthias Riemenschneider, Steffi Neubauer, Katharina Seck, Hanns-Georg Klein, Marion Kiechle, Florian Lordick, Alfons Meindl

**Affiliations:** 1 Department of Gynecology, Klinikum rechts der Isar, Technische Universität München, München, Germany; 2 Center of Human Genetics and Laboratory Medicine, Martinsried, Germany; 3 Department of Psychiatry and Psychotherapy, Klinikum rechts der Isar, Technische Universität München, München, Germany; 4 Department of Medical Oncology, National Center for Tumor Diseases, Heidelberg, Germany; Deutsches Krebsforschungszentrum, Germany

## Abstract

**Background:**

Cancer patients carrying mutations in the dihydropyrimidine dehydrogenase gene (*DPYD*) have a high risk to experience severe drug-adverse effects following chemotherapy with fluoropyrimidine drugs such as 5-fluorouracil (5-FU) or capecitabine. The pretreatment detection of this impairment of pyrimidine catabolism could prevent serious, potentially lethal side effects. As known deleterious mutations explain only a limited proportion of the drug-adverse events, we systematically searched for additional *DPYD* variations associated with enhanced drug toxicity.

**Methodology/Principal Findings:**

We performed a whole gene approach covering the entire coding region and compared *DPYD* genotype frequencies between cancer patients with good (n = 89) and with poor (n = 39) tolerance of a fluoropyrimidine-based chemotherapy regimen. Applying logistic regression analysis and sliding window approaches we identified the strongest association with fluoropyrimidine-related grade III and IV toxicity for the non-synonymous polymorphism c.496A>G (p.Met166Val). We then confirmed our initial results using an independent sample of 53 individuals suffering from drug-adverse-effects. The combined odds ratio calculated for 92 toxicity cases was 4.42 [95% CI 2.12–9.23]; *p (trend)*<0.001; *p (corrected)* = 0.001; the attributable risk was 56.9%. Comparing tumor-type matched sets of samples, correlation of c.496A>G with toxicity was particularly present in patients with gastroesophageal and breast cancer, but did not reach significance in patients with colorectal malignancies.

**Conclusion:**

Our results show compelling evidence that, at least in distinct tumor types, a common *DPYD* polymorphism strongly contributes to the occurrence of fluoropyrimidine-related drug adverse effects. Carriers of this variant could benefit from individual dose adjustment of the fluoropyrimidine drug or alternate therapies.

## Introduction

5-fluorouracil (5-FU) and orally available 5-FU prodrugs remain a backbone of chemotherapy for locally advanced and metastatic gastroesophageal, colorectal, and breast cancer [1]–[5], but can result in toxic effects. Severe and unpredictable drug-adverse events are mainly attributed to deficiency of the enzyme dihydropyrimidine dehydrogenase (DPD). Due to its function as initial and rate-limiting enzyme in the catabolism of pyrimidines, DPD deactivates more than 80% of administered standard doses of 5-FU [6]–[8]. The impairment of this pyrimidine degradation pathway leads to toxic accumulation of the drug and, most likely, concerns also patients treated with 5-FU-prodrugs like capecitabine [9]. Estimating a frequency of 3–5% of patients harbouring at least a partial DPD deficiency, the pretherapeutical detection of this metabolic dysfunction could prevent severe and unwanted side effects due to fluoropyrimidine drugs.

After the characterization of the highly polymorphic human dihydropyrimidine dehydrogenase gene (*DPYD*, MIM# 274270) [10], rapid genetic testing has become feasible and numerous sequence aberrations have been found in different ethnic populations [11]–[16]. Specific *DPYD* variants result in a truncated protein with clear deleterious effect to the enzyme including the exon-14-skipping mutation IVS14+1g>a which has been considered as the most prevalent mutation in DPD deficient patients [17]. However, such truncating mutations have appeared to explain only a limited number of serious side effects attributed to DPD deficiency. [18]–[20]. Moreover, only few missense mutations are known to directly interfere with protein structure, cofactor binding or electron transfer of the DPD enzyme (e.g. c.703C>T; c.2846A>T) [21]–[23]. Up to now, the impact of (common) non-synonymous polymorphisms on fluoropyrimidine-induced toxicity remains widely unclear and systematic association studies are therefore mandatory.

In this context, the sequence variation c.496A>G (p.Met166Val) has been classified either as a mutation which is related to DPD deficiency [24], [25] or as a variant accompanied with normal DPD activity in peripheral blood cells [26]. Here we are presenting data showing a high prevalence of the c.496G risk allele in cancer patients with increased toxic reactions compared to patients with good tolerance of a fluoropyrimidine-containing chemotherapy.

## Methods

### Objectives

In this study, we thoroughly evaluated the risk of several genetic variants covering the entire *DPYD* gene for association with enhanced toxicity during standard fluoropyrimidine-based chemotherapy.

### Participants

Our initial patient sample (n = 128) consisted of Caucasian subjects that had been diagnosed for breast, gastroesophageal and colorectal cancer between 2003–2006 (Table 1) and who received treatment with 5-FU-based therapy regimens (Table S1, supporting information) at the Klinikum rechts der Isar, Technische Universität München; the Klinikum Hamburg-Eppendorf and at other institutions in Germany.

**Table 1:** Patient characteristics.

	CONTROLS	CASES WITH HIGH TOXICITY	
		Cohort 1	Cohort 2
Number of subjects:	89	39	53
Mean age at diagnosis:	58.2+/−10.9	61.4+/−10.2	62.2+/−9.6
**Gender:**			
Male	56	19	25
Female	33	20	28
**Tumor:**			
Colorectal	15	14	35
Gastroesophageal	58	16	8
Breast	14	9	6
Not specified	2	0	4
**Chemotherapy regimen:**			
PLF+/−Paclitaxel	38	10	1
OLF/FOLFOX	13	5	14
FOLFIRI	0	0	7
Mayo Protocol	1	5	1
5-FU/RTX	19	9	2
CMF	3	5	0
FEC	11	4	3
Xeloda+/−Oxaliplatin	0	0	14
Other/not available*	4	1	11
**Toxicity (NCI-CTC-AE):**	Grade 0–II	Grade III–IV	Grade III–IV
Lethal outcome	0	2	0

* Chemotherapy contained 5-FU, but other components not specified.

Abbreviations: PLF, cisplatin, 5-fluorouracil, folinic acid; OLF/FOLFOX, oxaliplatin, 5-fluorouracil, folinic acid; Mayo protocol, 5-fluorouracil, folinic acid; FOLFIRI, irinotecan, 5-fluorouracil, folinic acid; CMF, cyclophosphamide, methotrexate, 5-fluorouracil; FEC, 5-fluorouracil, epirubicin, cyclophosphamide.

For replication of our results, we included an additional cohort of 53 cancer patients who had been independently genotyped (*DPYD* exons 2, 6, 13 and 14) by the Center of Human Genetics and Laboratory Medicine, Munich-Martinsried, Germany, during 2005–2007 because of acute drug-adverse reactions (Table 1, cohort 2; and Table S1). Among these subjects, 14 had received therapies containing the orally available 5-FU prodrug capecitabine (Xeloda, Hoffmann LaRoche Pharma, Switzerland, [27]).

In addition, a previously analyzed cohort of 157 healthy individuals without a background of cancer [28] was considerably enlarged up to 607 volunteers and genotyped for the variants c.496A>G (rs2297595) and IVS10-15t>c.

### Ethics

Written informed consent had been obtained from all participating subjects and the study had been approved by the local Ethics Committee.

### Toxicity assessment

Side-effects that are typically associated with 5-FU treatment, like neutropenia, thrombopenia, mucositis, diarrhea, nausea and vomiting, neurotoxicity, cardiac toxicity, alopecia and hand-foot-syndrome were documented within the first 3 cycles of the therapy. The toxicity assessment was based on the National Cancer Institute Common Toxicity Criteria Adverse Event reporting guidelines (NCI-CTC AE, version 3.0) and was done without knowledge of the genotyping results. No distinct therapy protocol appeared to be particularly linked to higher unwanted toxicity.

### PCR amplification and mutational analysis

DNA was prepared from frozen EDTA-blood samples using standard techniques. The entire coding region of the *DPYD* gene was amplified with 23 primer pairs corresponding to 23 exons and the exon-intron-boundaries [28]. The detection of *DPYD* sequence variants was carried out by denaturing HPLC analysis and sequencing as previously described [29].

### Statistical methods

The pairwise linkage disequilibrium measures, D′ and r^2^, were calculated using the software package Haploview [30].

Association between affection state (5-FU tolerance) of the patient sample and genotypes was tested by logistic regression analysis including age at treatment and sex as covariates. Different single-marker association models were tested: no specific inheritance model (considers all genotypic effects), the recessive model, which contrasts one homozygote against the other pooled genotypes (both alleles as recessive alleles were tested) and a trend model which assumes an allele dose effect. Corrections for multiple comparisons within each model were considered using a global permutation test (1000 permutations). In addition, a two-marker sliding window approach was performed to narrow down the association signal.

Fisher's exact test was applied to assess differences in the distribution of polymorphisms with respect to toxicity in tumor-type matched pairs of patient samples.

The proportion of the risk of side effects in the 496A>G carriers that could be attributed to the G-allele (attributable risk in the “exposed”) was calculated by the following formula: attributable risk % (AR%) = (I_e_−I_u_) / I_e_×100, where ‘I_e_’ is the incidence of side effects in the combined group of heterozygous and homozygous carriers of the G-allele and ‘I_u_’ is the incidence of side effects in the group with the major genotype (AA).

## Results

### Characteristics of the initial patient cohort

Based on common toxicity criteria guidelines (NCI-CTC AE, version 3.0), we obtained a total of 39 cancer patients presenting with grade III and IV toxicity after treatment with a 5-FU-containing (poly)chemotherapy (Table S2, supporting information). Thirty-seven of these individuals showed recovery from the encountered adverse events following complete elimination of 5-FU or corresponding dose reductions. Two patients had a fatal outcome: One 62 year-old patient (Table S2, patient #26) with rectal cancer developed severe neutropenia, mucositis and diarrhea after 5-FU administration and died to the sequela of a toxic shock syndrome. Another 76 year-old man (patient #17) with locally advanced adenocarcinoma of the esophagogastric junction died during the first 5-FU/oxaliplatin application. He had reported severe angina pectoris and suffered a cardiac arrest. Immediate resuscitation remained unsuccessful. Although autopsy did not reveal structural damages of coronary arteries or heart muscles, his death was attributed to 5-FU-induced cardiac toxicity, possibly due to coronary spasm. In the majority of our recruited patients (n = 89), however, 5-FU-based chemotherapy was well tolerated or caused only mild toxicity (NCI-CTC AE grading I–II).

### Association of distinct polymorphisms with enhanced 5-FU-induced toxicity

**Figure 1:**
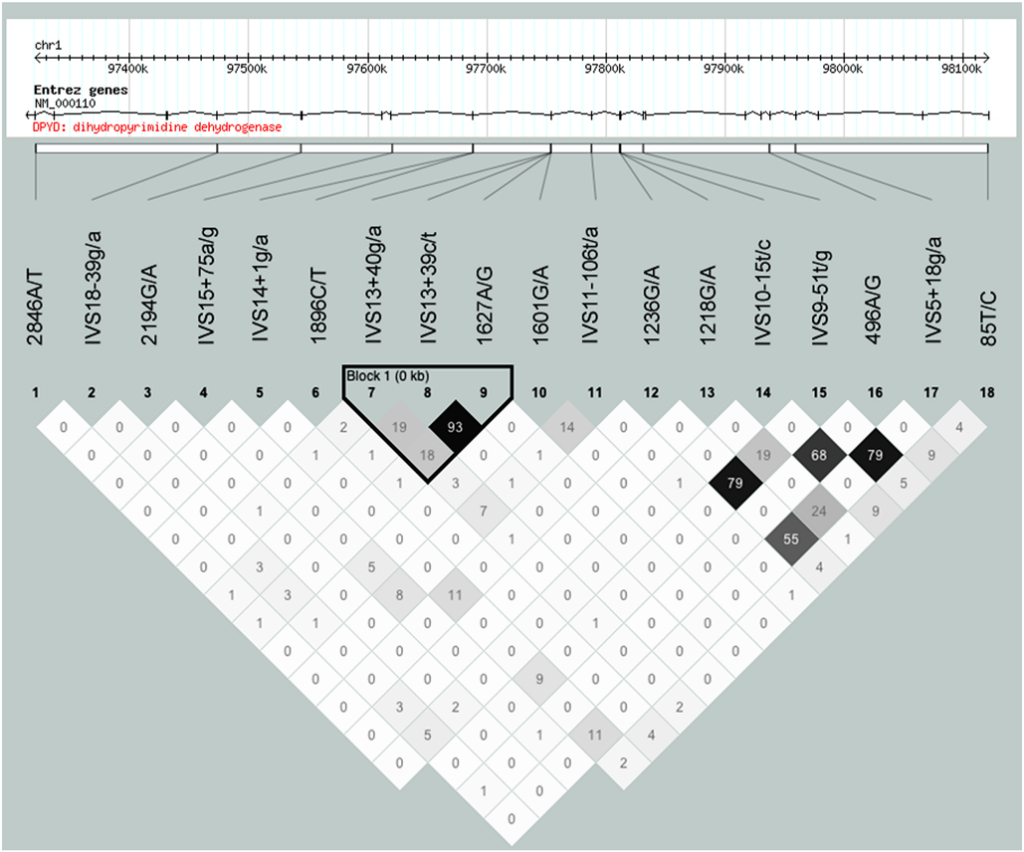
Linkage disequilibrium (LD) structure based on 18 *DPYD* variants. Pairwise LD measures (r^2^) calculated with the software package Haploview [ref. 30] are shown. The strongest LD region is highlighted.

Scanning the entire reading frame of the *DPYD* gene in the above described patient cohort, we identified 18 different single nucleotide exchanges and one novel frameshift mutation, c.1109delTA (p.Ile370LysfsX4), distributed across the entire *DPYD* gene (Table 2). The linkage disequilibrium (LD) structure based on 18 SNPs shows considerable low D′ and r^2^ values with only two genetic regions showing moderate LD (Fig. 1). This D′ and r^2^ pattern may indicate a large recombination-rich DNA interval comprising the complete *DPYD* gene as suggested before [31], [32].

**Table 2:** Allele frequencies of *DPYD* variants in patients with/without enhanced toxicity.

Variation^1^/ rs-number	Effect	Minor allele frequency				Association with high toxicity
		Healthy	Patient controls	Cohort 1	Cohort 2	
		individuals	(no/mild toxicity)	(toxicity III–IV)	(toxicity III–IV)	
		n = 157^2^	n = 89	n = 39	n = 53^3^	
c.85T>C / rs1801265	p.Cys29Arg	0.19	0.25	0.28	0.26	
IVS5+18G>A	-	0.01	0.01	0.03	n.a.^4^	
**c.496A>G / rs2297595**	**p.Met166Val**	**0.08 (n = 607)**	0.08*^§^	0.26*^§^	**0.23^§^**	* OR = 4.56 [1.95–10.75]; ***p(corr)*** ** = 0.002**
						**^§^OR = 4.42 [2.12–9.23]; ** ***p(corr)*** ** = 0.001**
IVS9-51T>G	-	0.02	0.02	0.03	n.a.	
c.1109delTA	frameshift	0	0	0.013	n.a.	
**IVS10-15T>C**	**-**	**0.11 (n = 453)**	0.085*	0.24*	n.a.	*OR = 3.88 [1.71–8.78]; ***p(corr)*** ** = 0.009**
c.1218G>A	p. Met406Ile	0.01	0.006	0	n.a.	
c.1236G>A	p. Glu412Glu	0.003	0.01	0.03	n.a.	
IVS11-106T>A	-	0.07	0.09	0.05	n.a.	
c.1601G>A / rs1801158	p.Ser534Asn	0.016	0.006	0.06	0.009	
c.1627A>G / rs1801159	p.Ile543Val	0.14	0.23	0.23	0.20	
IVS13+39C>T	-	n.a.	0.24	0.22	0.20	
IVS13+40G>A	-	n.a.	0.39	0.34	0.33	
c.1896T>C / rs17376848	p.Phe632Phe	0.04	0.04	0.05	0.05	
IVS14+1G>A	exon14 deletion	0	0	0.03	0.03	
IVS15+75A>G	-	0.17	0.17	0.17	n.a.	
c.2194G>A / rs1801160	p.Val732Ile	0.02	0.05	0.05	n.a.	
IVS18-39G>A / rs12137711	-	0.11	0.10	0.09	n.a.	
c.2846A>T	p.Asp949Val	0.006	0	0.013	n.a.	

1 Reference sequences are based on NCBI Accession No. NM_000110.3 (mRNA).

2 Published in Seck et al, 2005 (Ref.28).

3 Cohort of patients analyzed by the Zentrum für Humangenetik und Laboratoriumsmedizin, Martinsried, Germany.

4 not analyzed.

Analysis of the *DPYD* genotypes revealed evidence of a strong allele-dose-dependent association with the appearance of toxicity for two moderately correlated variants, IVS10-15t>c and c.496A>G (r^2^ = 0.68; *p (trend)*<0.001), even after adjustment for multiple testing (IVS10-15t>c: *p (corrected)* = 0.009, odds ratio (OR) = 3.88 [95% confidence interval 1.71–8.78]; c.496A>G: *p (corrected)* = 0.002; OR = 4.58 [1.95–10.75]; Table 2). Regarding the two-marker sliding window approach the strongest association signals were observed for those haplotype combinations which included the marker c.496A>G (e.g. marker combination c.496A>G and IVS5+18 g>a: haplotype G/G; frequency in patients with and without toxicity; 0.256 vs. 0.088; *p* = 0.0003). This suggests that the single marker association signal is mainly due to the functional SNP c.496A>G causing a non-synonymous amino acid substitution (p.Met166Val) at a highly conserved position and within a conserved three-dimensional environment [25], [33].

In the studied patient cohort, the attributable risk to suffer from severe drug-adverse effects due to the 496G-allele was 56.9%. Further clinical support of the relevance of this polymorphism with respect to enhanced toxicity may come from the patient samples: one case of cardiac death concerned a heterozygous G-allele carrier (patient #17, Table S2) and all homozygous carriers of the G-allele (patients #16, 23, 31) fell into the subgroup of patients with enhanced toxicity. The considerable lower frequency of the c.496G-allele in patients with good tolerance of 5-FU (0.082) was identical with the population-based control group (0.081) consisting of 607 healthy individuals (Table 2).

### Reevaluation of the association data with an additional patient cohort

To corroborate our findings we included a second cohort of 53 patients which has been collected and analyzed independently (cohort 2, Table 2). All patients had reacted with severe drug-adverse events following treatment with a 5-FU- or capecitabine-based chemotherapy regimen. The c.496A>G minor allele frequency of this second cohort (0.23) showed no relevant difference compared to the initial toxicity group (0.26). In this context it is interesting that three c.496G allele carriers encountered severe toxicity (mainly diarrhea and hand-foot-syndrome) after application of the orally available 5-FU prodrug capecitabine, which resulted in cessation of the chemotherapy in two individuals. These observations may emphasize a risk for drug intolerance due to DPD involvement in chemotherapy regimens using capecitabine [9].

Analysis of the combined patient sample with toxicity (initial and second cohort; n = 92) yielded a significant dose-dependent association for the *DPYD* marker c.496A>G (*p (trend)*<0.001; *p (corrected)* = 0.001; OR = 4.42 [2.12–9.23]. In addition, no significant association with fluoropyrimidine-related side effects was observed for the other *DPYD* polymorphisms, gender and age at treatment.

### Incidence of c.496A>G in tumor type-matched sets of samples

Since distinct DPYD polymorphisms could be correlated with a particular type of tumor, we additionally re-evaluated our association data in tumor-type matched sets of patients (Table 3). Gastroesophageal and breast cancer patients reflected the results obtained in toxicity and control cases of the whole patient population. No association of c.496A>G or IVS10-15t>c with enhanced toxicity was achieved for colorectal carcinoma cases, although a trend towards higher prevalence of these variants was linked to side effects following a fluoropyrimidine/platinum therapy (e.g. c.496A>G frequency in toxicity versus control group: 0.29 versus 0.17; *p* = 0.378).

**Table 3:** Comparison of control and toxicity cases in tumor-type matched patient samples.

**Gastro-Esophageal Cancers**					
	**Grade 0–II**		**Grade III–IV**		
SNP	Alleles	Total	Alleles	Total	*p*-value*
c.496A>G	6	110	10	48	0.007
IVS10-15T>C	8	114	8	32	0.008
**Colorectal Cancers**					
	**Grade 0–II**		**Grade III–IV**		
SNP	Alleles	Total	Alleles	Total	*p*-value
c.496A>G	6	30	22	98	not significant
IVS10-15T>C	5	30	5	28	not significant
**Breast Cancers**					
	**Grade 0–II**		**Grade III–IV**		
SNP	Alleles	Total	Alleles	Total	*p*-value
c.496A>G	2	26	11	30	0.013
IVS10-15T>C	2	28	6	18	0.014

* Comparison of frequencies in toxicity grade 0–II versus III–IV using Fisher's exact test.

### Low frequency of clear deleterious mutations in our patient population

The well-described exon-14-skipping mutation IVS14+1g>a which is related to DPD deficiency occurred in only five of all 92 cases with toxic side effects. This splice-site mutation was not observed in patients with good tolerance of a fluoropyrimidine therapy. Another yet undescribed truncating mutation (c.1109delTA) was discovered in a patient who suffered fatal toxicity during the 1^st^ cycle of 5-FU monotherapy (patient #26, Table S2). The previously unknown frameshift mutation in exon 10 leads to a stop codon at position 374. Finally, the missense mutation 2846 A>T (p.Asp949Val), which is assumed to interfere with iron-sulfur-cluster formation and thus, with the electron transfer during the catalytic reaction of the enzyme DPD [21], was only found in one individual with severe enterotoxicity of grade IV (patient #29, Table S2).

## Discussion

Sequence variations in the *DPYD* gene have been shown to influence the breakdown of the common anticancer drug 5-FU and to provoke severe drug-adverse effects during systemic 5-FU-application in cancer patients. Moreover, the integrity of the 5-FU degradation pathway appears to be of similar importance concerning the application of newly introduced fluoropyrimidine drugs which are intracellularly converted into 5-FU [9], [34], [35]. Thus, these observations warrant systematic detection of DPD-deficient patients prior to fluoropyrimidine administration. However, a practical and reliable pretreatment test for *DYPD* variants or mutations has not been available so far due to the high genetic variability of the *DPYD* coding region and the rare occurrence of clear deleterious mutations, at least in Caucasian populations [18], [19]. For this reason, several functional methods designed for the rapid prediction of a (partial) DPD deficiency such as the 2-^13^C-uracil breath test [36] or the determination of plasmatic uracil/dihydrouracil ratios [34], [37], [38] have been introduced in the meantime. Mercier and colleagues reported very recently, that prospective evaluation of the functional DPD status followed by corresponding 5-FU dose tailoring led to a 2- fold decrease in the occurrence of severe toxicities [39]. Nevertheless, these methods have not found broad application in clinical routine so far [40], not least because these kind of analyses require a special equipment. In addition, a lack of correlation between DPD activity measurements and 5-FU toxicity was assumed [41]. Clearly, methodologies based on genetic testing for clinically relevant SNPs would offer the simplest way to identify patients at the highest risk of potentially life-threatening drug-adverse events.

With respect to the development of a genetic test, we conducted a systematic analysis of the coding region of the gene *DPYD* and compared the incidence of commonly found SNPs between cancer patients with good and with poor tolerance of a fluoropyrimidine-based chemotherapy. We observed a significant allele-dose-dependent association of the non-synonymous sequence aberration c.496A>G (p.Met166Val) with the phenotype of enhanced toxicity of grade III/IV. The methionine-valine exchange resulting from the c.496A>G transition has been already implicated in a deleterious effect in DPD deficient patients [24], [25], but conflicting results have been reported for its influence on enzyme activity [26]. While DPD activity measured in peripheral blood mononuclear cells might be unrelated to 5-FU toxicity according to a study by Di Paolo et al. [41], the high conservation of the mutation site during evolution strongly speaks in favour of a biological relevance of this amino acid change [33]. Most strikingly, carriers of the c.496A>G genotype constituted more than 43% of the individuals with severe drug-adverse effects in our study. In contrast to this high prevalence, the classical exon-14-skipping mutation IVS14+1g>a (DPYD*2A) occurred in only five patients (5.4%) of overall 92 toxicity cases. Moreover, another yet undescribed truncating mutation, c.1109delTA (p.Ile370LysfsX4), and a putative deleterious missense mutation (c.2846A>T) were detected each once in the studied patient population. Thus, compared to other recent publications which reported either marginal predictive potential [19], [20] or a reduction of severe adverse effects of up to 27% by prospective genotyping for the mutations IVS14+1g>a and/or c.2846A>T [40], [15], the detection of a more frequent polymorphism associated with an elevated risk for fluoropyrimidine intolerance would help to identify much more risk patients. These individuals could benefit from careful individual dose adaptation of 5-FU or 5-FU prodrugs.

A potential bias of our study could have been introduced by association of c.496A>G with a certain tumor type. In this context, no discrepancy with results obtained in the whole patient population was obvious regarding breast or gastroesophageal cancers. In addition, the incidence of c.496A>G in the respective control cases without enhanced toxicity did not exceed the frequency measured in 607 healthy individuals speaking against a relation of c.496A>G with the development of these cancers. In contrast to these results, no significant correlation of c.496A>G with toxicity could be determined for colorectal cancer patients yet because the sequence aberration displayed increased incidence (compared to healthy volunteers) in toxicity as well as in control cases. However, due to the rather small number of control cases (n = 15) which were available for this type of cancer, the amount of 496G-allele carriers in the control group might have been overestimated and needs further evaluation with higher case numbers.

On the other side, the different results obtained with colorectal cancer patients could explain the discrepancy between our findings and those of Schwab et al. [20] concerning a major role of c.496A>G in severe drug-adverse effects. The recent study by Schwab et al. which suggested a limited role of genetic factors for severe 5-FU toxicity relies mainly on patients with colorectal carcinoma. Another reason for the different observations may be linked to the type of treatment of the patients, as Schwab et al. have restricted their clinical trial to 5-FU monotherapy. Whereas only 19% of gastroesophageal cancers were treated with 5-FU+/−folinic acid in our study, 45% of colorectal cancer patients received such treatment. Accordingly, less pronounced association with the variant 496A>G was found – although at low case numbers - for 5-FU alone or with chemoradiation yielding 496G-allele frequencies of 0.14 (n = 11) versus 0.105 (n = 19) in cancer patients with and without severe drug-adverse events. However, a high prevalence of the c.496A>G genotype was confirmed in our patients with severe toxicity regarding 5-FU/ platinum or anthracycline-containing regimens (frequency of 496G-alleles in patients with and without severe toxicity: 0.25 (n = 30) versus 0.08 (n = 51) for 5-FU/folinic acid/platinum drug therapies; 0.43 (n = 7) versus 0.05 (n = 10) for FEC treatment). These data may suggest that the influence of c.496A>G is more obvious in the presence of additional, drugs.

Since relatively high DPD activity has been reported for c.496A>G carriers by Johnson et al. [26] we cannot rule out that the severe c.496A>G-associated phenotype is due to a cumulative effect caused by toxic fluoropyrimidine catabolites [42] and cytotoxicity of other components of the polychemotherapy [43]. Nevertheless, our data obtained on a high number of toxicity cases (n = 92), comparable to the study by Schwab et al., show a clear, clinically important association which reached high significance in gastroesophageal and breast cancers.

With respect to the complexities in pharmacogenomics [44], evaluation of different therapy regimens and tumor types may lead to a better understanding of the role of genetic factors in fluoropyrimidine-related drug-adverse-events. Gene chip analyses for the detection of relevant *DPYD* variants as previously introduced by Zhang et al. [45] might then be the best choice in a future clinical setting.

### Limitations

Although this initial study relies on a relatively high number of patients with severe toxicity, case numbers are still limited. Analysis of further cases with toxic side effects is now utterly required taking also into account the specific type of tumor and treatment protocol.

## Supporting Information

Table S1 Treatment protocols with respect to the type of cancer
(0.04 MB DOC)

Table S2 Patients with grade III–IV toxicity, completely analyzed in the DPYD gene
(0.10 MB DOC)
