# Supplementary material for: Strong Association of a Common Dihydropyrimidine Dehydrogenase Gene Polymorphism with Fluoropyrimidine-Related Toxicity in Cancer Patients
Source: PLoS One. 2008 Dec 23;3(12):e4003. doi: 10.1371/journal.pone.0004003 (PMC2602733; doi:10.1371/journal.pone.0004003)
Supplement: Table S1 — Treatment protocols with respect to the type of cancer (0.04 MB DOC) [file pone.0004003.s001.doc]

**Table S1.** Treatment protocols with respect to the type of cancer

| **Gastro-Esophageal Cancers:** |  |
| --- | --- |
| PLF: | Cisplatin 50mg/m^2^ by intravenous (i.v.) infusion for 1 hour (h) on day 1, leucovorin 500mg/m^2^ 2h i.v. on days 1 and 8 and 5-FU 2000mg/m^2^ 24h i.v. on days 1 and 8. Repeated on day 15. |
| T-PLF: | Paclitaxel 85mg/m2 3h on day 1, cisplatin 50mg/m^2^ by intravenous (i.v.) infusion 1 h on day 1, leucovorin 500mg/m^2^ 2h i.v. on days 1 and 8 and 5-FU 2000mg/m^2^ 24h i.v. on days 1 and 8. Repeated on day 15. |
| OLF: | Oxaliplatin 85mg/m^2^ 2h i.v. on day 1, leucovorin 500mg/m^2^ 2h i.v. on days 1 and 8 and 5-FU 2000mg/ mg/m^2^ 24h i.v. on days 1 and 8. Repeated on day 15. |
| **Colorectal Cancers:** |  |
| 5-FU-chemoradiation  (5-FU/RTx): | 5-FU 250mg/m^2^/24h continous i.v. infusion during radiation treatment : 1.8 Gray (Gy) per day up to a total dose 45Gy. |
| Mayo regimen: | Leucovorin 20mg/m^2^ bolus on days 1 to 5 followed by 5-FU 425mg/m2 bolus on days 1 to 5. Repeated on day 29. |
| FOLFOX 4(6): | Oxaliplatin 85 mg/m^2^ on day 1; leucovorin 200 (350) mg/m^2^ on days 1 and 2, followed by 5-FU 400 mg/m^2^ bolus. Repeated on day 15. |
| FOLFIRI: | Irinotecan 180 mg/m^2^ 2h i.v. on day 1, leucovorin 400mg/m^2^ 2h i.v. on days 1 and 2 and 5-FU 400-500 mg/m^2^ bolus on days 1 and 2. Repeated on day 15. |
| XELOX: | Oxaliplatin 130mg/m^2^ on day 1, Capecitabine 1000 mg/m² on day 1 to 15 twice a day, repeated on day 22 |
| **Breast Cancer:** |  |
| CMF: | Cyclophosphamide 100mg/m^2^ per os (p.o.) on days 1 to 14, methotrexate 40mg/m^2^ i.v. on days 1 and 8, 5-FU 600mg/m^2^ i.v. on days 1 and 8; Repeated on day 29. |
| FEC: | 5-FU 500mg/m^2^ i.v. on day 1, epirubicine 100mg/m^2^ i.v. on day 1 and cyclophosphamide 500mg/m^2^ on day 1. Repeated on day 22. |
| Xeloda | Capecitabine 1250mg/m² per os (p.o.) on days 1 to 14 twice a day; repeated on day 22 |
