# Supplementary material for: Strong Association of a Common Dihydropyrimidine Dehydrogenase Gene Polymorphism with Fluoropyrimidine-Related Toxicity in Cancer Patients
Source: PLoS One. 2008 Dec 23;3(12):e4003. doi: 10.1371/journal.pone.0004003 (PMC2602733; doi:10.1371/journal.pone.0004003)
Supplement: Table S2 — Patients with grade III–IV toxicity, completely analyzed in the DPYD gene (0.10 MB DOC) [file pone.0004003.s002.doc]

**Table S2.** Patients with grade III-IV toxicity, completely analyzed in the DPYD gene

| ID | Tumortype | Age | Gender | Chemotherapy | 5FU-Dose (mg/m^2^) | Toxicity (NCI CTC AE grading) |
| --- | --- | --- | --- | --- | --- | --- |
| 1 | Breast | 49 | f | CMF | 600 | leukopenia (4), febrile neutropenia (3) |
| 2 | Rectum | 71 | f | 5FU/RTX | 250 | neutropenia (4), mucositis (3) |
| 3 | Breast | 29 | f | FEC | 500 | febrile neutropenia (4), anemia, thrombopenia, stomatitis (2) |
| 4 | Colon | 58 | f | Mayo | 425 | prolonged and pronounced mucositis and diarrhea (3) |
| 5 | Breast | 64 | f | CMF | 500 | diarrhea (4), neutropenia (3), central neurotoxicity (3) |
| 6 | Oropharynx | 70 | f | 5FU/RTX | 250 | Enhanced toxicity (3-4), not specified |
| 7 | Breast | 45 | f | CMF | 600 | mucositis (3), polyneuropathy (3) |
| 8 | Rectum | 42 | m | 5FU | 1000 | leukopenia and thrombopenia (4), mucositis (4), diarrhea (3) |
| 9 | Rectum | 71 | m | Mayo | 425 | diarrhea (4), vomiting (3), thrombopenia(2) |
| 10 | Stomach | 67 | m | Machover | Not specified | dermatitis (4), diarrhea (3) |
| 11 | Stomach | 61 | m | T-PLF | 2000 | diarrhea (3), vomiting (2) |
| 12 | Oropharynx | 63 | f | PLF | 2000 | mucositis (3) |
| 13 | Breast | 61 | f | CMF | 600 | leukopenia and thrombopenia (4), mucositis and diarrhea (3) |
| 14 | Breast | 53 | f | FEC | 500 | leukopenia (4), febrile neutropenia (4) |
| 15 | Breast | 62 | f | FEC | 500 | Enhanced toxicity (3), not specified |
| 16 | Breast | 76 | f | CMF | 600 | mucositis, vomiting, diarrhea (3), leukopenia and thrombopenia (2) |
| 17 | Esophageal junction | 76 | m | OLF | 2000 | cardiac death during cont. 5-FU infusion  likely due to coronary spasm |
| 18 | Rectum | 69 | m | Mayo | 425 | diarrhea and vomiting (3), hand-foot (2) |
| 19 | Colon | 55 | f | Mayo | 425 | mucositis and diarrhea (3), hand-foot (2) |
| 20 | Stomach | 66 | f | PLF | 2000 | diarrhea and vomiting (3), |
| 21 | Esophagus | 46 | m | 5FU/ RTX | 250 | mucositis (3), hand-foot (2) |
| 22 | Colon | 52 | m | OLF | 2000 | prolonged and pronounced angina pectoris (4) |
| 23 | Esophagus | 66 | m | PLF | 2000 | diarrhea (3) |
| 24 | Esophagus | 52 | m | T-PLF | 2000 | mucositis (3), diarrhea (2) |
| 25 | Rectum | 64 | m | OLF | 2000 | diarrhea (3), leukopenia and thrombopenia (2) |
| 26 | Rectum | 62 | m | 5FU | 2000 | fatal outcome after neutropenia (4), diarrhea (4), mucositis (4), cardiac arhythmia, coma, |
| 27 | Rectum | 75 | f | 5FU/RTX | 250 | diarrhea (3) |
| 28 | Stomach | 64 | m | PLF | 2000 | dermatitis (4) |
| 29 | Esophagus | 68 | m | PLF | 2000 | diarrhea (4) |
| 30 | Esophagus | 68 | m | OLF | 2000 | mucositis (4), dermatitis (3) |
| 31 | Breast | 56 | f | FEC | 500 | diarrhea and vomiting (3), pancytopenia (4) |
| 32 | Colon | 71 | f | OLF | 2000 | diarrhea (3-4) |
| 33 | Esophageal junction | 48 | m | T-PLF | 2000 | nausea and diarrhea (3) |
| 34 | Esophagus | 71 | m | PLF | 2000 | vomiting (3), diarrhea (2), angina pectoris |
| 35 | Colon | 60 | f | 5-FU | 950 | neuropathy (3), mucositis (2) |
| 36 | Stomach | 60 | m | T-PLF | 2000 | diarrhea (3), leukopenia (2) |
| 37 | Rectum | 70 | f | 5-FU | 500 | diarrhea (3) |
| 38 | Stomach | 71 | m | T-PLF | 2000 | diarrhea (3) |
| 39 | Stomach | 62 | m | T-PLF | 2000 | diarrhea (3) |
